# Extracellular matrix-associated proteins form an integral and dynamic system during *Pseudomonas aeruginosa* biofilm development

**DOI:** 10.3389/fcimb.2015.00040

**Published:** 2015-05-13

**Authors:** Weipeng Zhang, Jin Sun, Wei Ding, Jinshui Lin, Renmao Tian, Liang Lu, Xiaofen Liu, Xihui Shen, Pei-Yuan Qian

**Affiliations:** ^1^Division of Life Science, The Hong Kong University of Science and TechnologyHong Kong, China; ^2^Department of Biology, Hong Kong Baptist UniversityHong Kong, China; ^3^State Key Laboratory of Crop Stress Biology for Arid Areas and College of Life Sciences, Northwest A & F UniversityYangling, China

**Keywords:** biofilm, matrix-associated proteins, iTRAQ, type three secretion system, *Pseudomonas aeruginosa* ATCC27853

## Abstract

Though the essential role of extracellular matrix in biofilm development has been extensively documented, the function of matrix-associated proteins is elusive. Determining the dynamics of matrix-associated proteins would be a useful way to reveal their functions in biofilm development. Therefore, we applied iTRAQ-based quantitative proteomics to evaluate matrix-associated proteins isolated from different phases of *Pseudomonas aeruginosa* ATCC27853 biofilms. Among the identified 389 proteins, 54 changed their abundance significantly. The increased abundance of stress resistance and nutrient metabolism-related proteins over the period of biofilm development was consistent with the hypothesis that biofilm matrix forms micro-environments in which cells are optimally organized to resist stress and use available nutrients. Secreted proteins, including novel putative effectors of the type III secretion system were identified, suggesting that the dynamics of pathogenesis-related proteins in the matrix are associated with biofilm development. Interestingly, there was a good correlation between the abundance changes of matrix-associated proteins and their expression. Further analysis revealed complex interactions among these modulated proteins, and the mutation of selected proteins attenuated biofilm development. Collectively, this work presents the first dynamic picture of matrix-associated proteins during biofilm development, and provides evidences that the matrix-associated proteins may form an integral and well regulated system that contributes to stress resistance, nutrient acquisition, pathogenesis and the stability of the biofilm.

## Introduction

Infections caused by bacterial biofilms, which are composed of microorganisms that attach to a surface, have emerged as a major public health concern. Biofilm development occurs in sequential processes in general: attachment (phase I), microcolony formation (phase II), maturation I (phase III), maturation II (phase IV), dispersal (phase V) (O'Toole et al., 2000; Waite et al., 2005). In a biofilm, cells are embedded in the extracellular polymeric substance (EPS), also known as the extracellular matrix. The extracellular matrix consists (hereafter referred as matrix) of nucleic acids, polysaccharides, lipids and proteins. Several studies (Hall-Stoodley et al., 2004; Flemming and Wingender, 2010; Colvin et al., 2011; Lewenza, 2013) have shown that polysaccharides and DNA in the matrix play important roles in biofilm development. For example, polysaccharides provide mechanical stability, mediate bacterial adhesion to surfaces and form a cohesive, three-dimensional network that connects and immobilizes biofilm cells. Compared to polysaccharides, information about matrix-associated proteins is limited. Matrix-associated proteins have been identified from some microorganisms, such as *Haemophilus influenzae* biofilm that contains over 200 proteins involved in cell motility and secretion (Gallaher et al., 2006). In a recent study, the role of extracellular matrix binding protein in *Staphylococcus aureus* biofilm formation was reviewed (Speziale et al., 2014). However, the dynamics of matrix-associated proteins during biofilm development have not been systematically studied, and their roles in biofilm development remain elusive.

Nutrient acquisition, stress resistance and pathogenesis are important processes associated with biofilm development. Biofilm development is largely affected by nutrients that are available in the environment. For example, specific L-amino acids are required for the formation of a tight microcolony as well as various cysticfibrosis-specific phenotypes of *Pseudonomas aeruginosa* PAO1 (Sriramulu et al., 2005). Nutrients such as sucrose, phosphate and calcium enhance biofilm formation of *Sinorhizobium meliloti* as their concentrations increase (Rinaudi et al., 2006). In addition, biofilm development is associated with enhanced resistance to environmental stresses such as oxidative stress, antibiotics and host immune response (Mah and O'Toole, 2001; Arciola et al., 2005; Zhang et al., 2013a). The mechanisms underlying these types of resistance have been attributed to the expression of biofilm-specific genes and phenotypic changes (Mah and O'Toole, 2001; Arciola et al., 2005; Zhang et al., 2013a). Moreover, biofilm development has also been associated with a range of infections, whereas polysaccharide components of the biofilm matrix play roles in pathogenesis and facilitate biofilm development in the host (Goller and Seed, 2010).

*P. aeruginosa* is a model organism for biofilm research in the laboratory (Stewart et al., 1993). In the present study, we investigated the dynamics of matrix-associated proteins in biofilm development by *P. aeruginosa* ATCC27853. *P. aeruginosa* ATCC27853 is a clinical strain that is frequently used in antimicrobial susceptibility testing (Fass and Barnishan, 1979), and its draft genome was sequenced in 2012 (Fang et al., 2012). The genetic and molecular bases underlying biofilm development by this bacterial strain remains largely unknown. Using iTRAQ-based proteomic analysis (Wiese et al., 2007) to quantify matrix-associated proteins isolated from *P. aeruginosa* ATCC27853 biofilms in phases I–IV, we discovered significant changes in protein related to nutrient metabolism, stress resistance and pathogenesis. Subsequently, we investigated gene expression, protein-protein interactions and the influence of gene mutations on biofilm development.

## Materials and methods

### Bacterial strains, culture media, and biofilm development

The bacterial strains and plasmids used in this study are listed in Supplementary Table 1. *Escherichia coli* JM109 was used for the cloning experiments, and *E. coli* 17-1 λ pir was used for the conjugation experiments. *P. aeruginosa* ATCC27853 was obtained from China General Microbiological Culture Collection (CGMCC). *P. aeruginosa* ATCC27853 was grown at 37°C in M9 broth or on M9 agar plates containing 50 μg mL^−1^ kanamycin. Biofilms were developed using a static model according to the method described by Waite et al. (2005) with modifications. Briefly, nitrocellulose filters (diameter, 47 mm; Millipore, Bedford, MA, USA) placed on M9 agar were incubated with 10^5^ CFU of bacterial culture at 37°C before monitored at different time points.

### Microscopic observation and characterization

Biofilms were stained with fluorescein isothiocyanate (FITC) (Sigma, Poole, United Kingdom). Microscopic observations were performed using a Zeiss LSM 510 CLSM (Carl Zeiss, Jena, Germany) equipped with detectors for FITC. Images were obtained using a 63×/1.4 objective, and signals were recorded in the green channel (excitation 488 nm, emission 522/32 nm). The stacked CLSM images were then analyzed for microbial cell density, biovolume, mean thickness, maximum thickness and coverage using the image quantification tool PHLIP34.

### Exopolysaccharide quantification

The concentration of exopolysaccharides in the biofilms was measured according to previously reported procedures (Myszka and Czaczyk, 2009). Biofilm samples were thawed on ice and centrifuged at 15,000 g for 20 min. The pellets were re-suspended in ~3 mL of a cold sulfuric acid solution (0.2 M sulfuric acid, pH 1.1), and the biofilm matrix was broken using a glass hand homogenizer tube and pestle before centrifugation at 15,000 g for 20 min. The resulting supernatant was collected and suspended in water, followed by the addition of 1.4 mL of 77% (v/v) H_2_SO_4_. Subsequently, the samples were mixed with 200 μL of 1% (w/v) cold tryptophan. Finally, they were heated at 37°C for 20 min, and the colored product was evaluated at OD_500_. Calibration curves were prepared using dextran solution.

### Genome annotation

The draft genome sequence of *P. aeruginosa* strain ATCC27853 was downloaded from the NCBI bacterial genome database. Putative reading frames were identified using Glimmer v.3.0 (Delcher et al., 2007). For the annotation, BLAST searches were performed against clusters of orthologous group (COG, Version 9.05) (Tatusov et al., 2001), KEGG (Kanehisa and Goto, 2000), CAZy (Cantarel et al., 2009) and *P. aeruginosa* strain PAO1 according to procedures reported in our previous work (Zhang et al., 2013a). We used prokaryote databases on local servers and used an *E*-value cut-off of <10^−5.^ As a considerable number of hypothetical proteins were identified, the annotation of selected proteins was confirmed by a manual BLAST search against the NCBI non-redundant database.

### Extraction of matrix-associated proteins

The matrix-associated proteins were isolated from the biofilms of 12, 24, 48, and 96 h respectively. Matrix-associated protein preparations were obtained by referring to classical methods described previously (Zhang et al., 1999; Liu and Fang, 2002). The “regular centrifuge” method was used with modifications. Briefly, the biofilms were scraped from the medium and re-suspended in cold 0.9% NaCl prior to incubation at 4°C for 2 h. After centrifugation for 30 min at 4000 g at 4°C, the supernatant was filtered through a 0.22-μm pore-sized filter to remove bacterial cell contamination. The supernatant was then precipitated by the addition of 3 times the volume of acetone. After storage at −20°C overnight, matrix-associated proteins were pelleted by centrifugation for 30 min at 4000 g at 4°C. The protein pellet was re-suspended in a buffer containing 8 M urea and 40 mM HEPES (pH 7.5).

### iTRAQ protein quantification analysis

Protein labeling and strong cation exchange fractionation were performed as described in our previous study (Han et al., 2013). Protein collected from each sample was purified using the ReadyPrep 2-D Cleanup Kit (Bio-Rad, Richmond, CA) and quantified using the RC-DC kit (Bio-Rad, Richmond, CA). Subsequently, 200 μg of protein from each sample was reduced, alkylated and digested with trypsin. The desalted peptides were labeled with four different iTRAQ compounds: 114, 115, 116, and 117 for matrix-associated proteins from biofilms of 12, 24, 48, and 96 h, respectively. After labeling, the samples were mixed, dried and fractionated using a strong cation exchange (SCX) column. The peptides were subjected to high-pressure liquid chromatography (HPLC) and the quadrupole time-of-flight (QSTAR XL) MS system.

Protein identification and quantification also were performed as previously described (Han et al., 2013). Briefly, raw MS/MS data were converted into non-deisotoped and deisotoped *pkl* files using Proteinlynx Global SERVER 2.2.5 (Waters Corp, Milford, MA, USA). The reporter mass (114–118 Da) was extracted from the non-deisotoped file, and the deisotoped files were replaced with the same mass. The combined *pkl* files were submitted to Mascot version 2.3.0 (Matrix Science, Ltd., London, UK) to search against translated forward and reverse *P. aeruginosa* ATCC27853 protein sequences. The minmium number of peptides for protein is 2. We removed peptide scores that is less than 95% confidence identification level. We also performed a “target-decoy” database search, and the false discovery rate (FDR) in this analysis was controlled as less than 1%. Because 99% of the identified proteins varied 1.4-fold between the three experimental replicates (technical replicates that started from peptide labeling), the cut-off for the identification of significantly changed proteins was set at 1.4, corresponding to a 99% confidence level in the quantification analysis.

### Quantitative PCR (qPCR)

RNA was extracted from 40 Petri dishes containing biofilms using the ALLPrep DNA/RNA Mini Kit (Qiagen, Hilden, Germany). To remove potential DNA contamination, the TURBO DNA-free TM Kit was applied (Applied Biosystems, Foster City, CA). Subsequently, RNA was reverse transcribed into first-strand cDNA using the SuperScript III First-Stand Synthesis SuperMix Kit (Invitrogen, Carlsbad, CA) and random hexamers. The qPCR reactions were conducted using the Kapa SYBR Fast qPCR Kit (Kapa Biosystems, Woburn, MA) on a Mx3000P qPCR System (Agilent Technologies, Palo Alto, CA). Each 20-μl qPCR reaction contained 10 μl of 2 × Master Mix, 1 pmol/μl of forward and reverse primers, and 0.5 μl of either standard or environmental sample. The cycling parameters were 5 min at 95°C, followed by 40 cycles of 15 s at 95°C, 15 s at 50°C and 15 s at 60°C. The 16S rRNA gene was used as an internal standard in qPCR, and the fold change of one target gene from different biofilm stages was calculated by normalizing its Ct values to the Ct values of the 16S rRNA gene. Three experimental replicates (technical replicates that started from cDNA synthesis) were performed. The primers used in this study are listed in Supplementary Table 2.

### Construction of mutant strains

Mutant strain construction was conducted following the procedure described in our previous study (Zhang et al., 2013b). Using Δ*oprD* as an example, the plasmid pDM4Gm^r^ was constructed by inserting SaI- and BglII-digested Gm^r^ fragments (amplified using Gm^r^-SalI-F/ Gm^r^-XhoI-R and pZL184 as a template) into the SalI site of pDM4. The resulting plasmid pDM4Gm^r^-Δ*oprD* was used to construct the *oprD* deletion mutant of *P. aeruginosa* ATCC27853. Upstream and downstream PCR products of *oprD* were amplified using primer pairs Δ*oprD*up-SalI-F/Δ*oprD*up-R and Δ*oprD*down-F/Δ*oprD*down-EcoRI-R, respectively. The upstream and downstream products were then linked together by overlap PCR and inserted into the SalI/EcoRI sites of pDM4Gmr to generate pDM4Gm^r^-Δ*oprD*. Equal amounts of late logarithmic-phase cultures of *E. coli* S17-1λ pir grown at 30°C with shaking and of strain ATCC27853 grown at 37°C without shaking were mixed and grown on LB agar. Cells from the agar plates were suspended in LB medium, and a strain with a single crossover event was selected on minimal medium containing 100 μg mL^−1^ Gm. Strains with double crossovers were counter-selected using LB agar plus 10% sucrose.

## Results

### Developmental processes of the *P. aeruginosa* ATCC27853 biofilm

**Figure 1:**
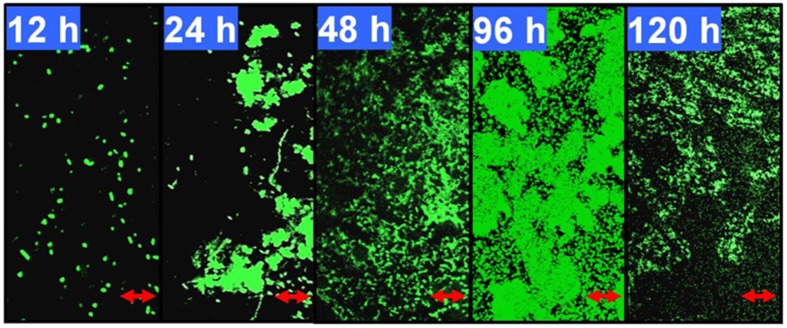
**Developmental processes of the *Pseudomonas aeruginosa* ATCC27853 biofilm**. Biofilms were developed using a static model according to the method described by Waite et al. (2005). The 12, 24, 48, 96, and 120-h-old biofilms were monitored by confocal laser scanning microscopy (CLSM). These five time points correspond to phases I–V, respectively, in this biofilm model. The scale bars are 10 μm. The coverage, thickness and biomass of the biofilms at different phases are shown in Supplementary Table 3.

As shown in Figure 1, after 12 h of growth on M9 medium, *P. aeruginosa* ATCC27853 cells remained separate but became aggregated and formed flat microcolonies with irregular shapes at 24 h. A large number of swimming bacteria were present in the first two phases of biofilm development. At 48 h, the cells became embedded in the matrix and complex three-dimensional structure began to form. At 96 h, the bacteria eventually formed multilayers, resulting in complex structures with a maximum thickness. Detachment was observed for biofilms that developed for 120 h. The coverage, thickness and biomass also were measured to confirm the phase classification (Supplementary Table 3). The biofilm reached its maximum biomass and coverage at 96 h. Therefore, in this model, growth for 12, 24, 48, and 96 h coincided with phases I–IV of biofilm development by *P. aeruginosa* ATCC27853, respectively.

### Comparative quantification of matrix-associated proteomes from different phases of biofilm development

**Figure 2:**
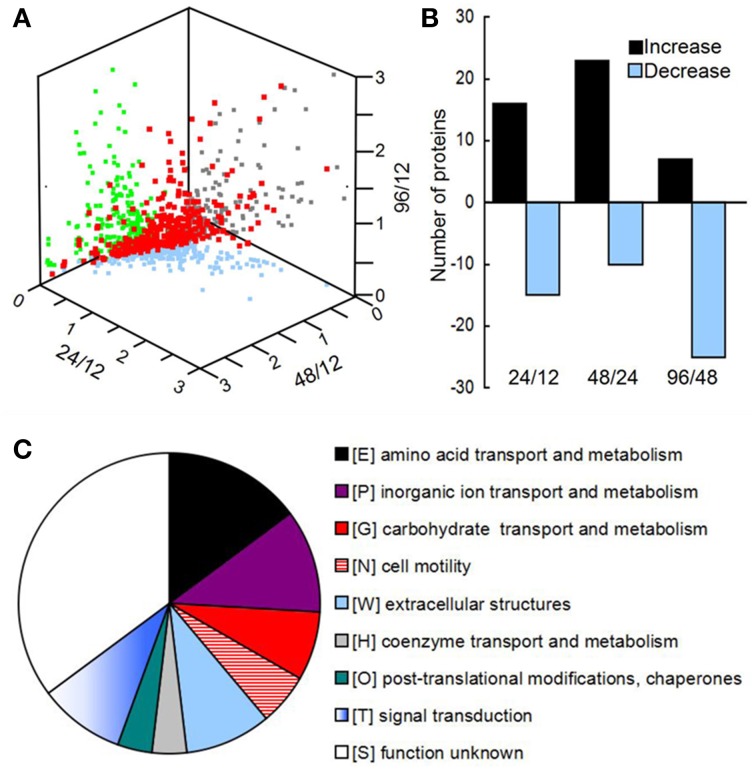
**(A)** Three-dimensional diagram representing the distribution of all identified proteins using the abundance ratios between different phases as the X-, Y-, and Z-axes, respectively. In total, 389 proteins were quantified, of which 54 showed significant changes among the different phases. **(B)** The numbers of proteins with different abundances from 12 to 24 h, 24 to 48 h, and 48 to 96 h. **(C)** Clusters of orthologous groups (COGs) of proteins with different abundances in the matrix during biofilm development.

Using OFFGEL fractionation of the pooled iTRAQ-labeled peptides and LC-MS/MS analysis, we identified 389 proteins (Supplementary File 1) from 5143 unique sequences. Comparison of the biofilm proteomes from different developmental phases revealed that 54 proteins had significantly different (≥q 1.4-fold) abundance (Figure 2A and Supplementary Table 4). From phase 12 to 24 h and 24 to 48 h, more proteins had an increased abundance than those with a decreased abundance; but from phase 48 to 96 h, most of the modulated proteins showed a decreased abundance (Figure 2B). Key protein structural components of the biofilm matrix were identified as CdrA and CupB5, which showed the highest abundance in phase I; this result was consistent with their roles in promoting biofilm formation and auto-aggregation (Borlee et al., 2010; Giraud et al., 2010). Moreover, the FlgM protein, which is secreted into the matrix in response to flagellar hook-basal body secretion (Guo et al., 2014), decreased by four-fold during biofilm development, which was consistent with the notion that motility is inhibited by biofilm development. The top 20 highly abundant proteins are listed in Supplementary Table 5, and most of them were secreted proteins such as lipoproteins and outer membrane proteins such as OmpA. All these results suggested that the method applied in this study to extract matrix-associated proteins was successful.

**Figure 3:**
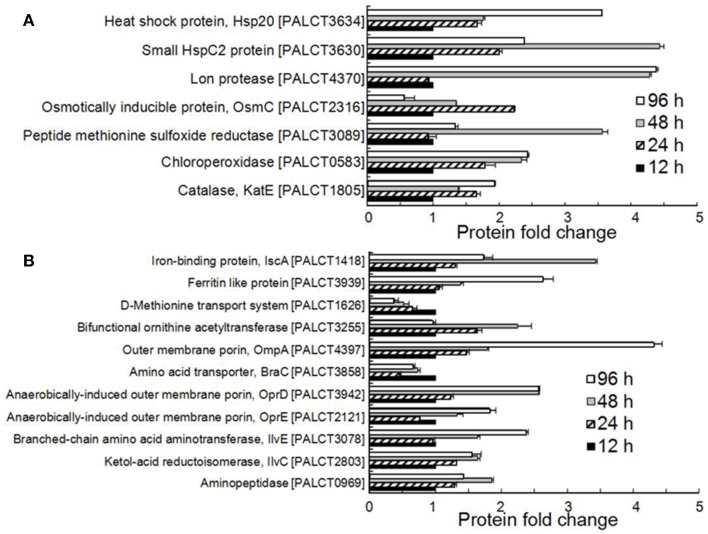
**Significantly changed proteins associated with stress resistance (A) and nutrient metabolism and acquisition (B) in the biofilm matrix**. Average values from three experimental replicates are shown.

All of the significantly changed proteins were grouped into several COG categories (Figure 2C and Supplementary Table 4). Proteins related to nutrient metabolism and acquisition changed in abundance in the matrix during biofilm development (Figure 3A and Supplementary Table 4). The category with the largest number of proteins was amino acid transport and metabolism, which consisted of Aminopeptidase, OmpA, IlvC, IlvE, OprD, and OprE, among others. Most of these proteins showed an increased abundance from phases I–IV. For example, the abundance of porin OmpA increased by four-fold during biofilm development. In addition to proteins classified under the category of amino acid transport and metabolism, other proteins related to nutrient acquisition also displayed significant changes, such as the iron-binding protein IscA.

Many proteins related to the stress response were identified and were significantly changed in abundance in the matrix (Figure 3B and Supplementary Table 4). These include enzymes responsible for resistance to mainly oxidative stress and heat shock stress. For example, the abundance of catalase (CatE), chloroperoxidase and methionine sulfoxide reductase increased during biofilm development. Catalases are H_2_O_2_ dismutases, chloroperoxidases are H_2_O_2_reductant oxidoreductases (Hewson and Hage, 2012), and methionine sulfoxide reductase is also required by bacteria to cope with oxidative stress (Sansom et al., 2013). In addition, the abundance of two heat shock proteins, the small HspC2 protein and Hsp20, also increased during biofilm development.

**Figure 4:**
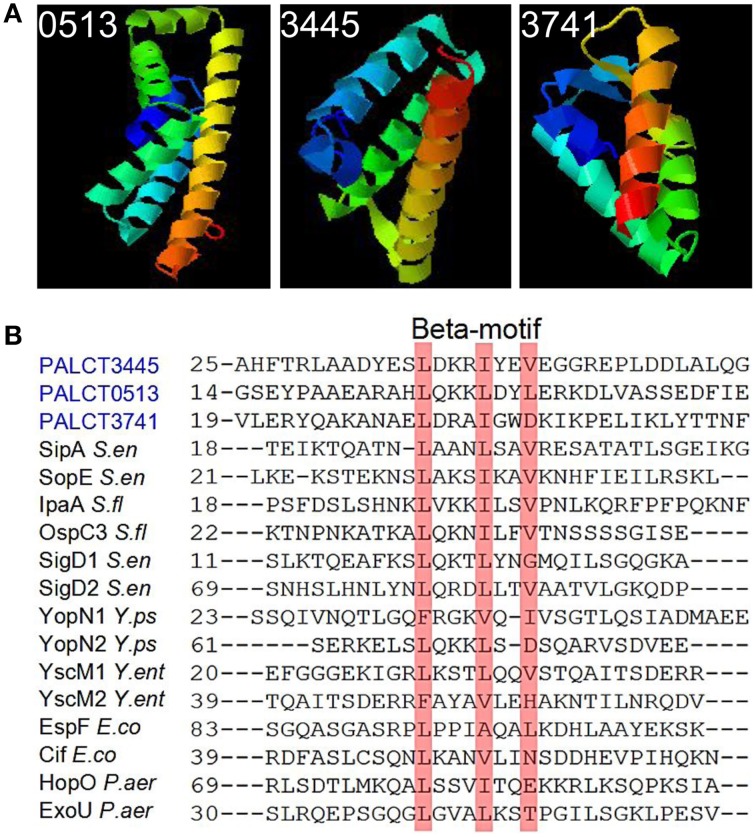
**Putative type three secretion system (TTSS) effectors identified in the biofilm matrix that also showed significant changes during biofilm development**. These proteins were identified based on signal peptide prediction by the Effective database. **(A)** The predicted protein structure was determined using QUARK software. **(B)** Conserved chaperone binding site in the three putative effectors and previously identified TTSS effectors.

Some of the changed proteins were secreted proteins related to pathogenesis, such as chitin-binding protein CbpD and elastase LasB, which increased in abundance in the late phases compared with the early phases of biofilm development. Efforts were taken to identify the putative functions of the hypothetical proteins by searching against known databases including COG, KEGG (version 67.0), CAZy and Effective (Jehl et al., 2001). As a result, three hypothetical proteins hits were found in the Effective database. Based on the detectable secretion signal for the type three secretion pathways, three proteins (PALCT0513, PALCT3445, and PALCT3741) were identified as putative effectors of the type III secretion system (TTSS). To obtain more supporting evidence, the three-dimensional structures of the three putative effectors were predicted using QUARK protein structure prediction software (Xu and Zhang, 2012). The three putative TTSS effectors displayed similar structures to known TTSS effectors (Figure 4A), which contain a signal peptide at the N-terminus and several helices (Lilic et al., 2006). Conserved beta motif-like sequences for chaperone binding also were identified based on sequence alignment with a previous study (Figure 4B) (Lilic et al., 2006).

### Expression profile of matrix-associated proteins

**Figure 5:**
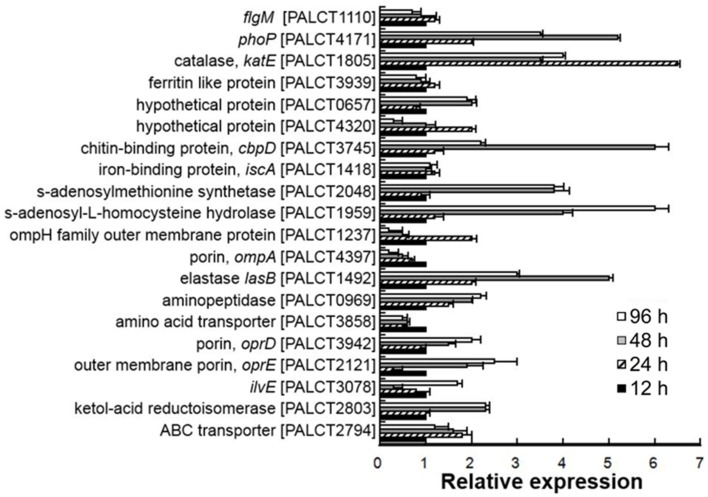
**Quantitative PCR analysis of selected genes encoding matrix-associated proteins in different biofilm phases**. The relative expression was calculated based on the ΔCt values. The 16S rRNA gene was used as a reference. Average values from three experimental replicates are shown. Correlation between the abundance of proteins and their corresponding gene transcripts is shown in Supplementary Figure 1.

As mentioned above, proteins related to nutrient metabolism, stress response and pathogenesis showed a significant change in abundance in the matrix during biofilm development. To determine whether these dynamics were consistent with the expression of the proteins, RNA was extracted from biofilms of 12, 24, 48, and 96 h, and the expression levels of 20 selected genes were compared using qPCR (see results in Figure 5). Among the examined 20 genes, the expression levels of five genes, including *lasB*, *phoP, ompA*, and *katE*, were not consistent with the protein expression levels. For the remaining 15 genes, there was a good correlation of the results in the proteomic analysis and the RT-PCR assay (Supplementary Figure 1). These findings suggested that the abundance of most matrix-associated proteins was affected by their expression levels and that the dynamics of matrix-associated proteins could be regulated by the bacterial cells.

### Protein-protein interaction networks and gene mutations

**Figure 6:**
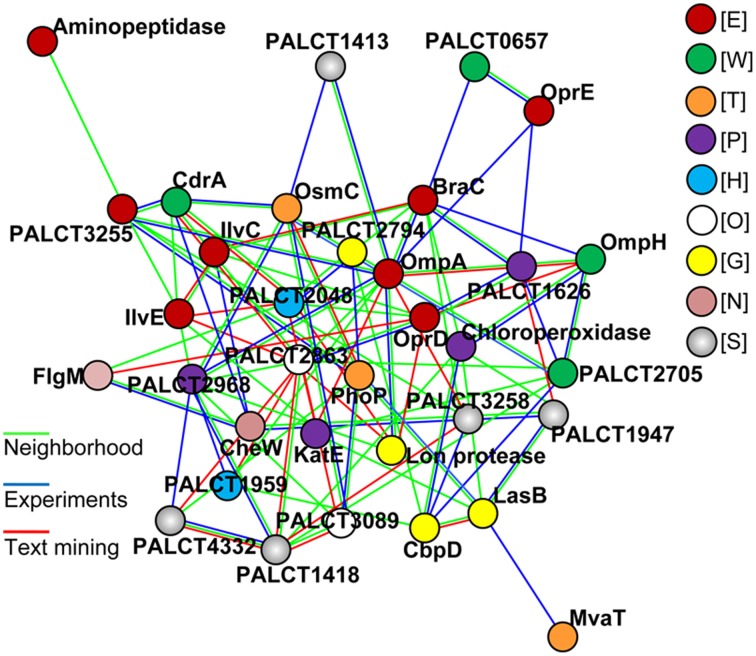
**The most significant protein-protein interaction model for proteins with a changed abundance in the matrix during biofilm development**. Lines in color indicate different interactions defined by the STRING. Proteins in color indicate different COG categories: E, amino acid transport and metabolism; W, extracellular structures; T, Signal transduction; P, inorganic ion transport and metabolism; H, coenzyme transport and metabolism; O, post-translational modifications, protein turnover, chaperones; G, Carbohydrate transport and metabolism; N, cell motility; S, function unknown.

Because several changed proteins in the matrix could be categorized under the same function (such as oxidative stress resistance), we speculated that these proteins interacted. Using the software STRING (Von Mering et al., 2003), an interaction network was built and shown in Figure 6; and many of these proteins were closely related to one another. Specifically, at the center of the network, OmpA interacted with OprE, OmpH, and Lon protease. The central network position of the OprD, OmpA, and CbpD proteins suggested that they exert important roles in *P. aeruginosa* ATCC27853 biofilms. To confirm this assumption, mutant strains of the three genes were obtained, and were compared with the wild-type stain. Indeed, the mutant strains CbpD and OmpA showed a decreased biofilm thickness, coverage and exopolysaccharide concentration at the maturation phase the OprD mutation affected the maximum thickness of the biofilm (Table 1).

**Table 1:** **CLSM-based characteristics of *P. aeruginosa* ATCC27853 and mutant biofilm structure in the mature II phase (phase IV, 96 h)**.

**Strains**	**Substrate coverage (%)**	**Average thickness (μm)**	**Maximum thickness (μm)**	**Exopolysaccharide (μg/10∧9CFU)**
Wild type	72.34 ± 2.12	6.98 ± 0.71	37.56 ± 3.54	32.45 ± 1.45
Δ*oprD*	65.36 ± 2.83	5.89 ± 0.50	24.12 ± 1.41^*^	30.47 ± 7.16
Δ*ompA*	34.79 ± 7.07^*^	3.28 ± 0.35^*^	19.54 ± 3.53^*^	14.67 ± 2.14^*^
Δ*cbpD*	45.87 ± 1.41^*^	4.31 ± 0.71^*^	14.56 ± 0.72^*^	12.39 ± 0.85^*^

“*” Indicates a significant change compared with the wild type strain.

## Discussion

Based on study of polysaccharides in the matrix, it has been hypothesized that the biofilm matrix is a dynamic environment in which microbial cells are optimally organized to use the available nutrients and resist stress (Hall-Stoodley et al., 2004; Flemming and Wingender, 2010). Here, we provide evidence to support this hypothesis based on the study of the dynamics of matrix-associated proteins. The results in the present study also suggest that the matrix-associated proteins, functioning as an integral system, contribute to pathogenesis and to the stability of the biofilm. Therefore, determination of the dynamics of matrix-associated proteins is a useful way to reveal their roles in biofilm development.

### Matrix-associated proteins and nutrient metabolism

The polysaccharides of the biofilm matrix act as an external digestive system by maintaining a close proximity between extracellular enzymes and bacterial cells, enabling them to metabolize dissolved, colloidal and solid organic carbons (Hall-Stoodley et al., 2004). Jiao et al. (2011) showed that natural biofilms contain CAZymes, which are likely to be involved in matrix degradation. One example is cellulase, which participates in the recycling of extracellular polysaccharides for nutrients or in biofilm dissolution. In the present study, we found that several proteins that accumulated in the matrix during biofilm development were related to nutrient metabolism, such as amino acid aminotransferase and aminopeptidase (Figure 3A). The increase in these proteins could be explained by biofilm growth-related stresses such as nutrient limitation. In addition to proteins related to amino acid metabolism, outer membrane proteins including transporters and porins also showed an increased abundance in the matrix from earlier to later phases. Outer membrane proteins are frequently identified in the extracellular space. The presence of these proteins in the matrix suggests that they may have roles in the biofilm other than nutrient transport. For example, it has been reported that OmpA is overexpressed in *Escherichia coli* during biofilm formation and that large quantities of OmpA can be secreted into the extracellular space (Gophna et al., 2004; Orme et al., 2006). OmpA is essential for biofilm development and resistance to antimicrobial agents in *Sodalis glossinidius* (Maltz et al., 2012). The *ompA* mutant strain fails to form biofilms *in vitro* and is unable to colonize the tsetse gut unless endogenous symbiotic bacteria are present (Maltz et al., 2012). In the present study, we consistently found that mutation of the *ompA* gene in *P. aeruginosa* ATCC27853 reduced the substrate coverage, thickness and concentration of exopolysaccharides. Collectively, the increase in nutrient metabolism-related and outer membrane proteins in the matrix may be a strategy for overcoming nutrient limitation and maintaining the stability of the biofilm. The reduced amounts of exopolysaccharides in the *ΔompA* biofilm also suggested a complex correlation between proteins and polysaccharides in the matrix.

### Matrix-associated proteins and stress resistance

Biofilm development is coupled to enhanced stress resistance. Polysaccharides, as the best studied components of the matrix, have been reported to provide protection against a variety of environmental stresses, including antibiotics, pH shifts, osmotic shock and desiccation (Hall-Stoodley et al., 2004; Flemming and Wingender, 2010). Here, we found that increased stress resistance during biofilm development also could be explained by the enhanced abundance of particular proteins in the matrix (Figure 3B). Catalase and chloroperoxidase, which act directly on peroxides, showed an increased abundance in the matrix. Based on previous studies (Stewart et al., 2000), catalases protect aggregated bacteria by preventing the complete penetration of hydrogen peroxide into the biofilm. CatE was identified as a component of the biofilm matrix formed by *P. aeruginosa* PAO1 (Toyofuku et al., 2012), and the abundance of catalase genes were found to be enriched in later compared to earlier phases of biofilm development in our previous study examining the intertidal biofilm community (Zhang et al., 2013a). Moreover, methionine sulfoxide reductase also increased during biofilm development. Methionine sulfoxide reductase has been demonstrated to play an important role in oxidative stress resistance (Lei et al., 2011), and it also reduces oxidized methionine in surface adhesion, thus facilitating biofilm formation under oxidative stress (Mintz et al., 2002). In addition to the oxidative stress-related proteins, proteins contributing to heat shock stress resistance were also increased in the matrix during biofilm development. Heat shock proteins have been reported to promote biofilm formation in several species, and they are up-regulated in biofilms compared to the planktonic state (Ito et al., 2009; Kuczyńska-Wiśnik et al., 2010). The actual function of heat shock proteins in the matrix could be to protect and stabilize proteins as chaperones; however, extracellular heat shock proteins are involved in the modulation of host immunity responses (Retzlaff et al., 1994). In a previous study on biofilms from acid mine drainage (Jiao et al., 2011), cold shock rather than heat shock proteins were identified in the biofilm matrix, suggesting that biofilms from different environments may take different strategies for stress resistance. All of these results suggested a correlation between increased stress resistance during biofilm development and the dynamics of matrix-associated proteins.

### Secreted proteins in the biofilm matrix

Secreted proteins are important parts of the biofilm matrix, and in the present study, several secreted proteins identified changed their abundance during biofilm development. Thus, the potential functions of secreted proteins in the matrix are discussed. One example is the elastase LasB, which is an important virulence factor and permits escape from phagocytosis (Kuang et al., 2011). Moreover, LasB can also influence the formation and architecture of mucoid *P. aeruginosa* SG81 biofilm as a result of changes in the matrix composition and properties (Tielen et al., 2010). Another secreted protein with a changed abundance in the matrix was CbpD, a chitin-binding protein. Chitin-binding proteins can enhance the rate of bacterial infection in human intestinal cells (Chaudhuri et al., 2010); chitin binding proteins also are involved in adaptation to environmental nutrient gradients, tolerance to stress and protection against predators (Pruzzo et al., 2008). Considering all of these findings, we hypothesize that the increased abundance of the two secreted proteins in the biofilm matrix may contribute to both pathogenesis and biofilm stability. Moreover, three secreted proteins in the matrix are likely to be novel effectors of the TTSS in the *P. aeruginosa* ATCC27853 biofilm and their abundance in the matrix decreased during biofilm development (Figure 4 and Supplementary Table 4). Effectors of the secretion system are powerful weapons used by bacteria to infect hosts (Hauck et al., 2003; Geddes et al., 2007). The increase in LasB and CbpD and decrease in putative effectors suggest that different virulence strategies are adopted by biofilms at different phases.

Many proteins with modulated abundance in the matrix were cytoplasmic proteins. This result was consistent with findings in a previous study (Jiao et al., 2011) that examined biofilm matrix-associated proteins. In addition, increasing evidence shows that many previously-known intracellular proteins such as catalase and heat shock proteins also exert functions outside of the cell. In a recent study (Foulston et al., 2014), Foulston et al. reported that the extracellular matrix of the *Staphylococcus aureus* biofilm comprises cytoplasmic proteins that associate with the cell surface in response to decreasing pH. While the presence of these proteins could be due to unknown secretion processes, another explanation is programmed cell death in the biofilm community, which naturally causes lysis (Bayle, 2007). The results represented in the present study suggested close interactions among the changed proteins; and mutation of the selected proteins in the center of the interaction network attenuated biofilm formation. In addition, based on expression profiling, the dynamics of matrix-associated proteins seem to be regulated by the bacteria. Furthermore, the functions and phenotype shifts observed during biofilm development were consistent with the dynamic patterns of the matrix-associated proteins. For example, as mentioned above, the enhanced stress resistance, nutrient acquisition and pathogenesis during biofilm development could be explained by the dynamic pattern of matrix-associated proteins. Taken together, we propose that the proteins in the matrix would be well organized and act as an integral part of the biofilm, whereas most of the proteins, including extracellular, outer membrane and cytoplasmic proteins, are functional components of the biofilm. The present study has elucidated potential roles of extracellular matrix-associated proteins in *P. aeruginosa* ATCC27853 biofilm development from a dynamic perspective, aiming to provide resources for the mechanistic understanding and control of bacterial biofilms. As an extension, our future focus will include interactions of particular proteins identified in the present work and their roles in biofilm development. For example, three secreted proteins in the matrix are likely to be novel effectors of the TTSS in the *P. aeruginosa*, and their functions await further characterization.

### Conflict of interest statement

The authors declare that the research was conducted in the absence of any commercial or financial relationships that could be construed as a potential conflict of interest.
