# Supplementary material for: Extracellular matrix-associated proteins form an integral and dynamic system during Pseudomonas aeruginosa biofilm development
Source: Front Cell Infect Microbiol. 2015 May 13;5:40. doi: 10.3389/fcimb.2015.00040 (PMC4429628; doi:10.3389/fcimb.2015.00040)
Supplement: Supplementary file 1 [file DataSheet1.PDF]

SUPPLEMENTARY INFORMATION

Extracellular matrix-associated proteins form an integral and dynamic system during *Pseudomonas aeruginosa* biofilm development

Weipeng Zhang[a]*, Jin Sun[b]*, Wei Ding[c], Jinshui Lin[c], Renmao Tian[a], Liang Lu[a], Xiaofen Liu[a], Xihui Shen[c], Pei-Yuan Qian[a#]

Division of Life Science, The Hong Kong University of Science and Technology, Kowloon, Hong Kong SAR, China[a]; State Key Laboratory of Crop Stress Biology for Arid Areas and College of Life Sciences, Northwest A & F University, Yangling, Shaanxi, China[c]; Department of Biology, Hong Kong Baptist University, Hong Kong SAR, China[b]

Running title: Dynamics of biofilm matrix-associated proteins

*These two authors contributed equally to this work

[#] Corresponding author:

Prof. Pei-Yuan Qian

Email: boqianpy@ust.hk

Telephone: +852 23587331

Fax: +852 23587323

Keywords: Biofilm; Matrix-associated proteins; iTRAQ; Type three secretion system; *Pseudomonas aeruginosa* ATCC27853

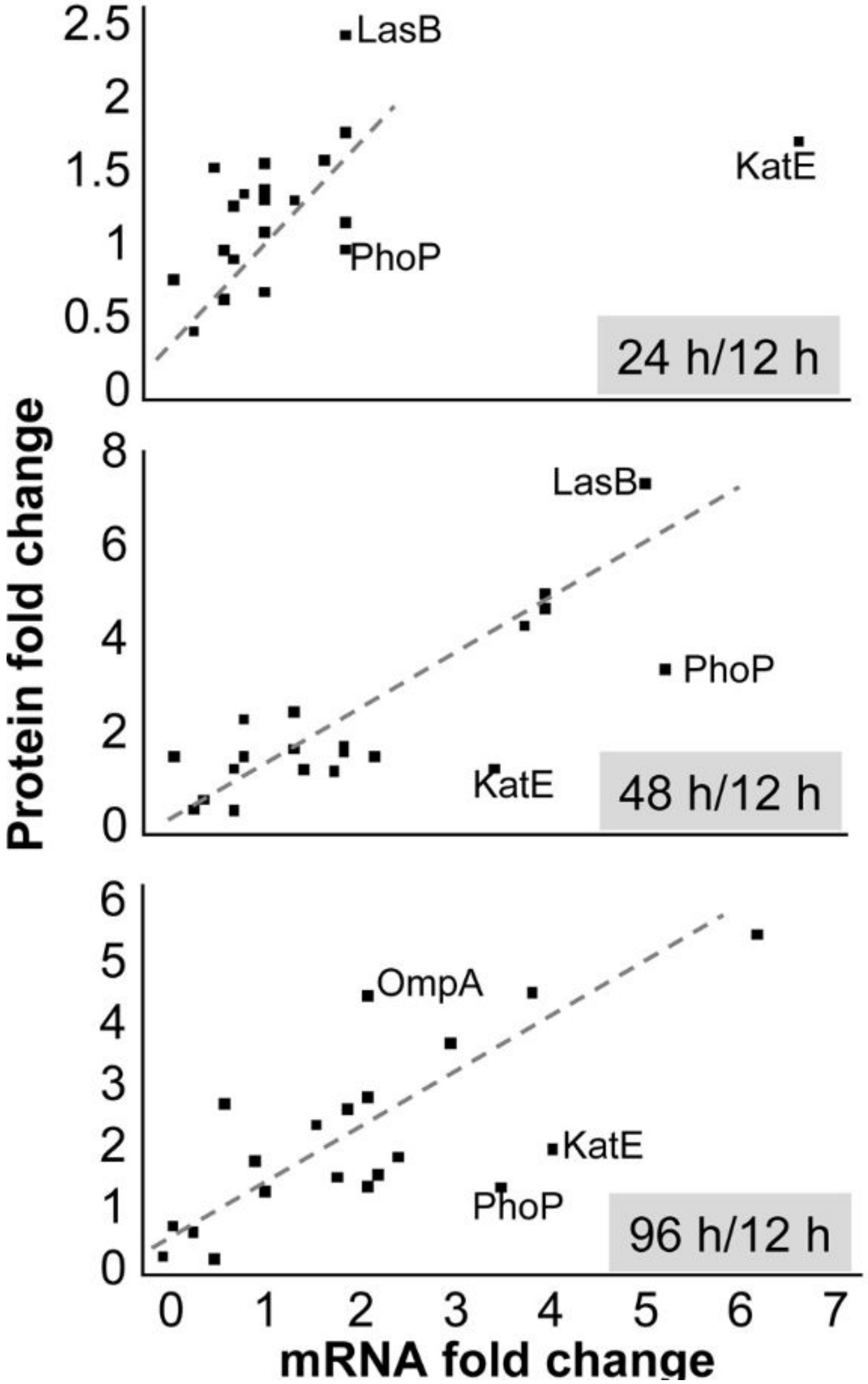

Supplementary Figure 1. Correlation between the abundance of matrix-associated proteins and their gene transcripts in biofilms from the phases I-IV. The fold change of mRNA of one target gene from different biofilm stages was calculated by normalizing its Ct values to the Ct values of the 16S rRNA gene.

Supplementary Table 1. Bacterial strains and plasmids used in this study.

**Strain or plasmid**	**Relevant characteristics**	**Reference**
E. coli S17-1*λpir*	*thi pro hsd* (r- m+) *recA::*RP4-2-Tcr::Mu Km^r::Tn7， lysogenized by λpir phage	Simon, *et al*. (1983)
pDM4	Suicide vector, mobRK2 oriR6K (pir requiring) sacBR of *Bacillus subtilis*, Cm^r	O' Toole, *et al*. (1996)
pDM4-Gm^r	pDM4 carrying Gm^r gene at *SalI* site	This study
pDM4-Gm^r-Δ*oprD*	pDM4 carrying 2 DNA fragments up- and downstream of *oprF*, Gm^r	This study
P. aeruginosa ATCC27853	Wild-type *P. aeruginosa ATCC27853*, Km^r	Fass, *et al*. (1979)
Δ*oprD*	*oprD* gene deleted in ATCC27853, Km^r	This study
Δ*ompA*	*ompA* gene deleted in ATCC27853, Km^r	This study
Δ*cbpD*	*cbpD* gene deleted in ATCC27853, Km^r	This study

Cm^r, Km^r and Gm^r represent resistance to chloramphenicol, kanamycin and ampicillin at 30, 50 and 100μg mL^{-1}, respectively.

Supplementary Table 2. Primers used in this study.

Name	5'-3' sequence
**RT-PCR**	
PALCT1110-F	TCAACCGGCTGAATCCCGGC
PALCT1110-R	AACCTTATCGTTGTCGACCA
PALCT2121-F	CGTGACCAAGGGCGAAGT
PALCT2121-R	CGGTTCAGGAACGGGAAGT
PALCT3939-F	TCAATATCGGAATCGGCGAA
PALCT3939-R	ATGCGCTCGGCGATGTCTCG
PALCT1959-F	TCGCCGAATCGGAAATGC
PALCT1959-R	CCAGGGTCTCGATCAGCAC
PALCT2048-F	CGACGAGAAGATCGTGCAACT
PALCT2048-R	GGATGACGACCGAAGTGGC
PALCT3745-F	GGCTGCTTCCTCGAAGGTCC
PALCT3745-R	TTGACGCCATTCCAGTCGTAC
PALCT3942-F	TAACCTCAGCGCCTCCCT
PALCT3942-R	TTGCCTTCATCGTTTGTGC
PALCT4171-F	TAACCTCAGCGCCTCCCT
PALCT4171-R	TTGCCTTCATCGTTTGTGC
PALCT4320-F	ACGACGGAAAACAACACCCC
PALCT4320-R	GAAAATTCGGCCTCCACCTC
PALCT1418-F	GAAGCCGCCGCCAAACAC
PALCT1418-R	CAGTCGGTGCCATCGAGA
PALCT0657-F	TCAAGACCGCCGAGACCA
PALCT0657-R	GGAGACACGCAGGAACGAG
PALCT2048-F	GAAGGCCATCCGGACAAG
PALCT2048-R	CGGCTTGGCCCGGTCGAC
PALCT0969-F	GTCGGTTCCTGTTCGCATTG
PALCT0969-R	GTCGGCGTAGTTCGGGTGT
PALCT4397-F	GCGTTGTCATCGGCTCGCT
PALCT4397-R	GGCGTCCAGGGAGGTCAG
PALCT1237-F	ACGGCAGCAAGATGTCCC
PALCT1237-R	CAGCTTCGGCTTGAGTTTC
PALCT1492-F	CCGACGACAGCAAGACCAC
PALCT1492-R	ACCAGTCCCGGTACAGTTTGA
PALCT2803-F	CGCTCACGGCTTCTCCAT
PALCT2803-R	CACCGCCCTTGACGAACT
PALCT3078-F	ACCCTGCACTATGGAATGGG
PALCT3078-R	CGAACAGCCGGTCGGTAT
PALCT3858-F	GCCTGTTCGCCGCGATGG
PALCT3858-R	CGTAGATCACGCCTTCGAG
PALCT1805-F	GGCAGGAGCAGCCTGACTT
PALCT1805-R	TCGTCGGTGAACAGATGGAA

PALCT2794-R	CTTGTAGGACTTGATCT
16S-F	CGCGAAGAACCTTACCTG
16S-R	ACCCAACATCTCACGACAC
**Mutation**	
Gm^{r}-*SalI*-F	GTGCGTCGACCACAGCACCTTGCCGTAGAAG
Gm^{r}-*XhoI*-R	GTGCCTCGAGTTAGGTGGCGGTACTTGGG
Δ*OprD*up-*SalI*-F	GTGCGTCGACCGGGTTGAAGAACTTTGAGG
Δ*OprD*up-R	TTGCCGGTTTCGTAGGTG
Δ*OprD*down-F	*CACCTACGAAACCGGCAA*AACGAGTACGGTGTAGAAGGTG
Δ*OprD*down-*EcoRI-R*	GTGCGAATTCCCCGAGCATACTGGAGCA
Δ*OmpA*up-*SalI*-F	GTGCGTCGACCGGGTTGAAGAACTTTGAGG
Δ*OmpA*up-R	CGCGGAACATCGGGTAGA
Δ*OmpA*down-F	*TCTACCCGATGTTCCGCG* GACCGAGCACGAAGAAGACAT
Δ*OmpA*down-*BglII-R*	GTGCAGATCTCGACGACATCCGCTACAACG
Δ*CbpD*up-*XhoI*-F	CTCGCTCGAGCCGCCGGCGCACCGGCAGATG
Δ*CbpD*up-R	CTCCGGACCTTCGAGGAAGCAGCCG
Δ*CbpD*down-F	*CGGCTGCTTCCTCGAAGGTCCGGAG*GTTCGATGCCCAGGGCCGC
Δ*CbpD*down-*BglII-R*	CTCGAGATCTTGATCGGCTGGATGCCGTAGCGGCT

Underlined nucleotides indicate restriction enzyme cutting sites that were added for cloning. Italics denotes mutation points used in overlap PCR assay.

Supplementary Table 3. The structural characteristics of *P. aeruginosa* ATCC27853 biofilm in the phases I-V based on confocal laser scanning microscopy (CLSM).

phase	**Substrate coverage (%)**	**Average thickness (μm)**	**Maximum thickness (μm)**	**Exopolysaccharide (μg/10^9CFU)**
I (12h)	6.79 ± 0.98	1.92 ± 0.31	3.61 ± 1.93	11.25 ± 3.28
II (24h)	45.32 ± 5.58	5.21 ± 0.77	14.52 ± 3.50	21.06 ± 5.31
III (48h)	64.32 ± 9.49	7.98 ± 2.65	35.44 ± 1.83	34.63 ± 4.21
IV (96h)	75.63 ± 5.68	8.67 ± 1.21	39.43 ± 5.64	36.34 ± 3.47
V (120h)	47.56 ± 3.28	5.22 ± 0.85	24.66 ± 1.17	27.54 ± 2.68

Supplementary Table 4. The matrix-associated proteins that were significantly changed during biofilm development.

Number	24 h/ 12 h	48 h/ 12 h	96 h/ 12 h	Protein	COG* Category	Changes
PALCT0969	1.27	**1.858**	**1.349**	Aminopeptidase	E	↑
PALCT2803	1.313	**1.638**	**1.534**	Ketol-acid reductoisomerase, IlvC	E	↑
PALCT3078	0.954	**1.64**	**2.308**	Branched-chain amino acid aminotransferase, IlvE	E	↑
PALCT2121	0.763	1.328	**1.813**	Anaerobically-induced outer membrane porin, OprE	E	↑
PALCT3942	1.235	**2.565**	**2.556**	OprD	E	↑
PALCT3858	**0.436**	0.731	**0.66**	Amino acid transporter, BraC	E	↓
PALCT4397	**1.478**	**1.789**	**4.298**	OmpA	E	↑
PALCT3255	**1.634**	**2.245**	0.934	Bifunctional ornithine acetyltransferase	E	↑↓
PALCT1626	**0.656**	**0.516**	**0.376**	D-Methionine transport system	P	↓
PALCT0583	**1.784**	**2.342**	**2.434**	Chloroperoxidase	P	↑
PALCT3939	1.064	1.378	**2.639**	Ferritin like protein	P	↑
PALCT1418	1.279	**3.429**	**1.741**	Iron-binding protein, IscA	P	↑↓
PALCT1805	**1.645**	1.379	**1.925**	Catalase, KatE	P	↑
PALCT2968	**1.464**	**3.22**	**1.795**	Bacterioferritin ion binding protein	P	↑↓
PALCT2794	**1.525**	1.365	1.285	ABC transporter	G	↑↓
PALCT1492	**2.325**	**7.324**	**3.569**	Elastase, LasB	G	↑↓
PALCT3745	**1.345**	**4.723**	**2.736**	Chitin-binding protein, CbpD	G	↑↓
PALCT4370	0.914	**4.284**	**4.344**	Lon protease	G	↑
PALCT4018	0.671	**4.625**	**6.753**	Purine-binding chemotaxis protein, CheW	N	↑
PALCT0621	1.346	**0.54**	**0.273**	FliZ	N	↓
PALCT1110	**0.685**	**0.497**	**0.235**	FlgM	N	↓
PALCT2705	**1.421**	**1.733**	**4.751**	LysM domain/BON superfamily protein	W	↑
PALCT0657	**0.639**	**1.721**	**1.5**	Putative outer membrane protein	W	↑
PALCT1237	1.131	**0.523**	**0.278**	OmpH family outer membrane protein	W	↓
PALCT3469	0.916	0.851	**0.372**	CdrA	W	↓
PALCT1752	0.756	**0.472**	**0.231**	CupB5	W	↓
PALCT2048	0.896	**4.356**	**4.348**	S-adenosylmethionine synthetase	H	↑
PALCT1959	**1.503**	**5.036**	**5.243**	S-adenosyl-L-homocysteine hydrolase	H	↑
PALCT2863	1.31	**2.45**	**1.752**	DNaK molecular chaperone	O	↑↓
PALCT3089	0.932	**3.567**	**1.316**	Peptide methionine sulfoxide reductase	O	↑↓
PALCT2316	**2.334**	1.347	**0.568**	Osmotically inducible protein, OsmC	T	↑↓
PALCT4171	0.957	**3.446**	**1.345**	Two-component response regulator, PhoP	T	↑↓
PALCT3203	**2.145**	**2.563**	1.372	Transcriptional regulator MvaT, P16 subunit	T	↑↓
PALCT3630	**1.889**	**4.435**	**2.328**	Small HspC2 heat shock protein	T	↑↓
PALCT3634	**1.656**	**1.759**	**3.559**	Heat shock protein, Hsp20	T	↑
PALCT1947	**0.448**	1.353	**0.553**	YceI-like domain protein	S	↓
PALCT1267	0.937	**0.321**	**0.557**	Hypothetical secreted protein	S	↓
PALCT3258	1.211	**3.453**	**3.92**	Hypothetical protein	S	↑↓
PALCT4332	**1.576**	0.964	**0.463**	Hypothetical protein	S	↓
PALCT1987	**0.591**	**0.541**	**0.283**	Hypothetical protein	S	↓

PALCT2044	**0.365**	**0.366**	**0.258**	Hypothetical protein	S	↓
PALCT0513	**0.626**	**0.645**	**0.463**	Hypothetical protein	S	↓
PALCT3741	**0.554**	**0.565**	**0.352**	Hypothetical protein	S	↓
PALCT3955	**0.438**	0.717	**0.57**	Hypothetical protein	S	↓
PALCT3950	1.308	**1.433**	1.233	Hypothetical protein	S	↑↓
PALCT4320	**1.703**	**1.632**	0.746	Hypothetical protein	S	↓
PALCT4655	**0.422**	**0.436**	**0.583**	Hypothetical protein	S	↓
PALCT0882	0.757	**0.643**	**0.476**	Hypothetical protein	S	↓
PALCT1360	**0.634**	**0.314**	**0.256**	Hypothetical protein	S	↓
PALCT1527	1.356	**1.434**	**2.244**	Hypothetical protein	S	↓
PALCT1413	**0.468**	0.849	**0.35**	Hypothetical protein	S	↓
PALCT3445	1.008	**0.613**	**0.323**	Hypothetical protein	S	↓
PALCT3166	**0.677**	**0.651**	**0.365**	Hypothetical protein	S	↓
PALCT2426	**0.787**	**0.425**	**0.334**	Hypothetical protein	S	↓

*COG categories: E, amino acid transport and metabolism; P, inorganic ion transport and metabolism; G, Carbohydrate transport and metabolism; N, cell motility; W, extracellular structures; H, coenzyme transport and metabolism; O, post-translational modifications, protein turnover, chaperones; T, signal transduction mechanisms; S, function unknown. Significant changes compared to phase I were highlighted in bold. The sign "↑" indicates an increase, the "↓" sign indicates a decrease, and the sign "↑↓" indicates a decrease after an increase, in protein abundance during biofilm development.

Supplementary Table 5. Top 20 proteins presented in the biofilm matrix of maturation phase (phase IV, 96h).

**Number**	**Annotation**
PALCT0682	Acyl carrier protein
PALCT3836	Flagellin and related hook-associated proteins
PALCT4237	ABC-type amino acid transport/signal transduction systems
PALCT3858	ABC-type branched-chain amino acid transport systems
PALCT3246	Chaperonin GroEL (HSP60 family)
PALCT2590	Putative translation initiation inhibitor
PALCT2756	Thiol-disulfide isomerase and thioredoxins
PALCT2847	Predicted periplasmic or secreted lipoprotein
PALCT1805	Catalase
PALCT3713	ABC-type amino acid transport/signal transduction systems
PALCT1984	Cold shock proteins
PALCT2863	Molecular chaperone
PALCT3334	Predicted periplasmic/secreted protein
PALCT3233	Superoxide dismutase
PALCT4533	Succinyl-CoA synthetase
PALCT1635	Hypothetical protein
PALCT3445	Uncharacterized protein conserved in bacteria
PALCT1422	Nucleoside diphosphate kinase
PALCT1395	ABC-type uncharacterized transport system
PALCT1867	Uncharacterized conserved protein
